# Dietary Capsaicin Protects Against Obesity via a Microbiome-Dependent Mechanism Independent of Host TRPV1 Signalling

**DOI:** 10.3390/nu18213667

**Published:** 2026-11-04

**Authors:** Ursula Amanda Sundoro, Jie Wang, Jing Luo, Amelia Ng, Weibiao Zhou, Hwei-Ee Tan

**Affiliations:** 1 Lee Kong Chian School of Medicine, Nanyang Technological University, Singapore 308232, Singapore; amanda.sundoro@ntu.edu.sg (U.A.S.);; 2 Department of Pathology, School of Basic Medical Sciences, Nanjing Medical University, Nanjing 211100, China; 3 Department of Food Science and Technology, National University of Singapore, Singapore 117542, Singapore

**Keywords:** host-knockout experimental framework, gut microbiota, dietary interventions, machine learning, food–microbiome interactions, prebiotic mechanisms, diet-induced obesity, lipid absorption

## Abstract

**Background/Objectives:** Dietary compounds simultaneously act on host tissue and gut microbiota, complicating efforts to distinguish direct host–receptor mechanisms from microbiome-mediated effects. Using a common food ingredient as proof-of-principle, this study aims to disentangle capsaicin host receptor signalling from capsaicin–microbiome interactions to determine the role of the gut microbiome in capsaicin-mediated metabolic protection. **Methods:** We employed a high-fat diet (HFD)-induced obesity model with or without capsaicin supplementation in TRPV1 knockout mice to determine the role of the host receptor. We also depleted the gut microbiota with antibiotics to determine the role of the microbiome. Host metabolic phenotypes were monitored, and microbiome 16S rRNA sequencing and metabolomics were analysed and extended with functional prediction and machine learning algorithms, followed by functional validation in vivo. **Results:** Capsaicin supplementation significantly protected against HFD-induced obesity, even capsaicin receptor knockout hosts. Crucially, depletion of the microbiota abolished this metabolic protection, demonstrating the microbiome’s obligatory role. While capsaicin supplementation did not alter hosts’ cumulative food intake, dietary preferences, or energy expenditure, it induced significant shifts in gut microbial diversity and function and triggered extensive faecal metabolomic remodelling which indicate altered nutrient processing including dampened lipid absorption. Consistent with this finding, in vivo functional validation also reveals attenuated circulating lipid response following oral fat administration, and this effect of capsaicin is abrogated without the microbiome. **Conclusions:** Although capsaicin is well-recognised as a modulator of host appetite and metabolism via canonical host receptor signalling, our strategic use of a host knockout has enabled us to unmask a nuanced microbiome-modulatory role of this chilli pepper ingredient: capsaicin tweaks the microbiome to potentially dial down host fat absorption and protect against high-fat diet-induced obesity. We also highlight a generalisable host knockout experimental framework for distinguishing microbiome-mediated pathways versus direct host receptor mechanisms.

## 1. Introduction

Chilli pepper (*Capsicum* spp.) is among the most widely consumed dietary ingredients worldwide. The principal bioactive component of chilli is capsaicin, which imparts chilli with its pungent and spicy characteristics. Besides contributing to a fiery flavour which is especially integral to some Asian and Latin American cuisines, capsaicin is also linked to numerous health benefits such as pain relief, reduced inflammation, improved cardiovascular and metabolic health [1,2,3]. Some evidence from human epidemiological and clinical studies indicates that capsaicin from chilli peppers may contribute modestly to weight management, including reducing body mass index, body weight, and waist circumference, though findings remain variable and controversial across diverse populations, cultural contexts, and study designs [4,5,6,7]. On the other hand, experimental animal studies have consistently demonstrated that capsaicin protects against high-fat diet-induced obesity and metabolic dysfunction [8,9,10,11,12,13,14,15,16,17].

The canonical mammalian receptor for capsaicin is the transient receptor potential vanilloid 1 (TRPV1), a molecular sensor for heat, acidity and inflammatory mediators [18,19]. TRPV1 is highly expressed in sensory neurons and is best known for its role in pain and heat sensation. However, TRPV1 expression has also been identified in multiple peripheral tissues including the intestine, pancreas, adipose tissue and brain, suggesting broader physiological functions beyond the sensory signalling [18,20]. Numerous studies have implicated TRPV1 activation in capsaicin’s effect against obesity: in adipose tissue, capsaicin-mediated TRPV1 activation has been reported to suppress adipogenesis and reduce lipid accumulation in white adipocytes [8,9,15]. In brown adipose tissue, TRPV1 signalling promotes energy expenditure through activation of thermogenic pathways [8,10]. Separate from potential appetite suppression stemming from capsaicin’s pungent sensation or pain, activation of TRPV1 in certain neurons in the brain has additionally been implicated in capsaicin’s effect on satiety [8,20]. Collectively, these observations have led to the prevailing model that capsaicin exerts its anti-obesity effects primarily through activation of host TRPV1 signalling pathways.

Concurrently, growing evidence indicates that diet-derived compounds are major determinants of microbial community structure and function within the gastrointestinal tract, and microbial metabolism profoundly influences host health and physiology through production of bioactive metabolites, modulation of bile acid pools, regulation of nutrient absorption and barrier function, and interactions with host immune and neuroendocrine pathways [21,22,23,24]. Recent studies have demonstrated that capsaicin supplementation alters gut microbial composition, improves glucose homeostasis, and modulates inflammatory responses [8,11,12,13,14,16,17,25,26]. Despite these findings, it remains unclear whether the metabolic benefits of capsaicin arise predominantly through direct activation of host TRPV1 signalling or indirectly through non-host mechanisms, such as microbiome modulation. Because dietary compounds simultaneously influence both host tissues and the gut microbiota, disentangling these effects causally is inherently challenging.

Our approach to addressing this question is the use of host receptor knockout models. If capsaicin retains anti-obesity activity in mice lacking TRPV1, this would suggest that the host TRPV1 signalling is not strictly required and that alternative mechanisms, including microbiome-dependent pathways, may contribute substantially to the observed metabolic phenotype. In this study, we investigated whether capsaicin suppresses high-fat diet-induced obesity independently of host receptor signalling and examined the potential contribution of the gut microbiome to this process. Using capsaicin receptor-deficient mice, microbiome profiling and perturbation, metabolomics and functional lipid assays, we identified a microbiome-dependent mechanism by which capsaicin reduces effective dietary fat absorption and attenuates weight gain during high-fat diet exposure.

## 2. Materials and Methods

### 2.1. Animals

**Figure 1:**
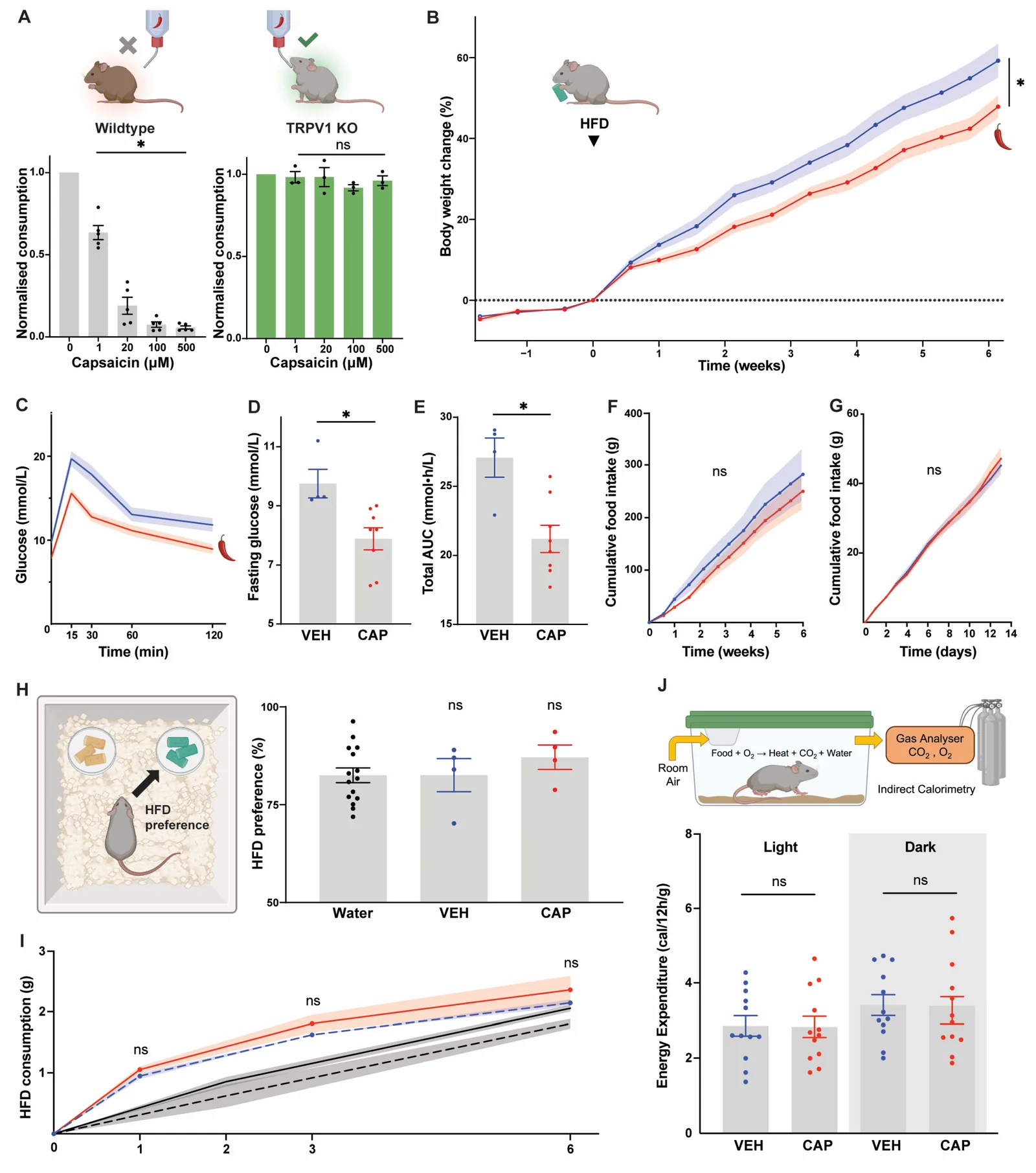
**Capsaicin ameliorates weight gain even in the absence of the host receptor.** (**A**) Consumption of capsaicin solution by wildtype (grey bars, *n* = 5) and TRPV1 KO (green bars, *n* = 3) mice normalised to vehicle intake. Significant aversion to capsaicin was observed in wildtype mice (*p* = 0.0012, one-way ANOVA), but not in TRPV1 KO at all concentrations (*p* = 0.20, one-way ANOVA). (**B**) Body weight changes in TRPV1 KO mice supplemented with capsaicin (red, *n* = 12) versus vehicle (blue, *n* = 12). Mice displayed rapid weight gain upon transition to obesogenic high-fat diet (HFD), compared to mice maintained on standard lower fat diet (Supplementary Figure S1A); however, obesity is significantly attenuated in the capsaicin group (*p* = 0.0171, two-way ANOVA). (**C**–**E**) Oral glucose tolerance test results, including blood glucose levels over time (**C**), glucose area under the curve (AUC, (**D**); *p* = 0.015), and fasting glucose levels ((**E**); *p* = 0.020; Welch’s *t*-test; CAP, *n* = 8; VEH, *n* = 4). (**F**,**G**) Cumulative HFD intake measured in standard cages ((**F**); *n* = 3 cages per group, 12 mice per group; *p* = 0.50) and a customised feeder setup ((**G**); *n* = 10 mice per group; *p* = 0.95; two-way ANOVA). (**H**) Innate preference for HFD compared to standard chow in naïve mice. All mice preferred HFD (>50%), and capsaicin (*n* = 4, *p* = 0.39, one-way ANOVA) or vehicle (*n* = 4, *p* > 0.99, one-way ANOVA) supplementation did not alter the preference compared to non-supplemented mice (*n* = 15). (**I**) HFD intake over time was assessed in capsaicin-supplemented (red solid line, *n* = 8) and vehicle-supplemented (blue dashed line, *n* = 8) mice that had been presented with 4 prior sessions of HFD tests. Note that the lines showing the rate of HFD consumption in experienced mice are much steeper compared to that of naïve mice (black lines, for reference), demonstrating strengthened preference for HFD after learning from experience. However, no significant difference in learned preference was found between the capsaicin and vehicle group (*p* = 0.26, two-way ANOVA). (**J**) Energy expenditure was measured in HFD-fed TRPV1 KO capsaicin-supplemented (red, *n* = 12) and vehicle-supplemented (blue, *n* = 12) mice by indirect calorimetry. Mice exhibit higher energy expenditure in the dark cycle (*p* = 0.028, two-way ANOVA) as anticipated due to greater activity, but capsaicin treatment does not alter energy expenditure compared to controls (*p* > 0.99 in light cycle, *p* > 0.99 in dark cycle, two-way ANOVA). Data is presented as mean ± SEM; ns, not significant; * *p* < 0.05.

All procedures were approved by and conducted in accordance with the Nanyang Technological University, Institutional Animal Care and Use Committee, Singapore. Male TRPV1 knockout mice (B6.129X1-Trpv1tm1Jul/J, #003770, The Jackson Laboratory, Bar Harbor, ME, USA) older than 7 weeks of age were used in all experiments [19]. For Figure 1A, wildtype (C57BL/6J, #000664, The Jackson Laboratory, Bar Harbor, ME, USA) mice were also used. Animals were housed in individually ventilated cages within specific pathogen-free barrier conditions at 22 ± 2° C and 50 ± 10% relative humidity, under a 12 h light-dark cycle, and maintained on standard facility chow (Altromin, Lage, Germany, 1324mod pellets, 11% kcal fat) until transitioned to high-fat diet (HFD, Research Diets Inc., New Brunswick, NJ, USA, D12492i pellets, 60% kcal fat), and on reverse osmosis purified water unless otherwise described.

### 2.2. Chemicals

For capsaicin supplementation [8], a solution of 61.2 µM capsaicin (Sigma-Aldrich, St. Louis, MO, USA, PHR1450) in 0.068% (*v*/*v*) ethanol (Sigma-Aldrich E7023) and 0.0767% (*v*/*v*) Tween-80 (Sigma-Aldrich P1754) in water was provided to mice to drink ad libitum unless otherwise indicated. Capsaicin and vehicle (0 µM capsaicin) supplementation commenced at least 1 week prior to the transition to HFD or start of preference tests.

In microbiome depletion experiments [27,28], a wide-spectrum antibiotic and antifungal cocktail containing 1 g/L ampicillin (VWR, Radnor, PA, USA, C0339-25G), 1 g/L neomycin (VWR C0558-25G), 0.5 g/L vancomycin (GoldBio, St. Louis, MO, USA, V-200-25), 0.2 g/L ciprofloxacin (GoldBio C-860-10), 1 g/L metronidazole (MP Biomedicals, Santa Ana, CA, USA, 155710) and 0.01 g/L amphotericin B (MedChemExpress, Monmouth, NJ, USA, HY-B0221) were given to mice to drink ad libitum, starting up to a month prior to the start of capsaicin or vehicle supplementation. Up to 600 mM sucrose (1st Base, Singapore, BIO1090) was initially added into the cocktail to motivate consumption and gradually weaned off. Sterility was confirmed through absence of growth from faeces in both aerobic and anaerobic cultures.

### 2.3. Capsaicin Sensitivity Test

After being singly housed and water-deprived from the previous day, TRPV1 KO and wildtype animals were provided with a bottle of capsaicin solution (either 0, 1, 20, 100, or 500 µM) for 3 h [19]. After an interval of 3 days, mice were water-deprived and tested again. The volumes consumed by each mouse were measured then normalised to the volume of 0 µM capsaicin solution that it had consumed.

### 2.4. Glucose Tolerance Test

HFD-fed mice on either capsaicin or vehicle were fasted for 12 h, before receiving 2 g/kg glucose (Sigma-Aldrich G7520) solution by oral gavage [12]. Immediately prior to (~0 min), and at 15, 30, 60, 120 min after glucose administration, blood samples were collected from the tip of the tail and glucose levels were measured with a glucometer (Abbott, Abbott Park, IL, USA, FreeStyle Optium Neo).

### 2.5. Feeding Assays

Food consumption was estimated by weighing the filled food hopper (~300 g) in standard cages (Tecniplast). To mitigate the variability due to relatively small food consumption per mouse per day, measurement intervals had to be extended to about twice a week (as plotted), and consumption was pooled by co-housing 4 mice per cage and then dividing the amount consumed by the number of mice in each cage to derive the average per mouse consumption for each cage.

We also developed an optimised feeder system engineered to fit within Tecniplast cages, in place of the original food hopper, that facilitates more sensitive per mouse measurements, and at daily intervals. In brief, a cage is partitioned into 2 halves with acrylic dividers, and a mouse is placed in each partition with individual food and fluid access slots. The custom stainless-steel feeders designed to fit in the food access slot contain a tray to collect fallen food crumbs, and a barrier to prevent animal or bedding interference. This mitigates variability due to fallen food crumbs. Further details including laser-cutting path design files and 3D sketches are provided in Supplementary Materials.

### 2.6. Preference Test

Singly housed 18 h fasted mice were presented with a Petri dish of HFD and a Petri dish of chow simultaneously for 6 h. HFD preference is calculated as the fraction of HFD to total food consumption [29]. Innate preference test was conducted for capsaicin-supplemented, vehicle-supplemented, and non-supplemented mice that were naïve to HFD. For learned preference experiments, capsaicin- and vehicle-treated cohorts were first given 4 sessions of HFD exposure as described above, and in the 5th session HFD intake was measured at timepoints from 0 to 6 h.

### 2.7. Energy Expenditure

Energy expenditure was assessed by indirect calorimetry using a PhenoMaster metabolic phenotyping system (TSE Systems). HFD-fed capsaicin-supplemented or vehicle-supplemented mice were individually housed in metabolic cages and acclimated for 48 h before data collection for more than 24 h according to manufacturer’s standard protocol. Energy expenditure was calculated from VO_2_ and VCO_2_ using the abbreviated Weir equation with CalR [30].

### 2.8. Microbiome Profiling

Faecal samples from 19 vehicle-supplemented and 19 capsaicin-supplemented mice were collected in sterile tubes, immediately snap-frozen in liquid nitrogen, and stored at −80 °C. The samples were then shipped to Biomarker Technologies (BMK, Beijing, China) on dry ice to ensure they remained frozen throughout transit. Upon arrival, DNA was extracted using the TGuide S96 DNA Kit (Tiangen Biotech, Beijing, China, DP812) according to the manufacturer’s instructions. The V3–V4 region of the 16S rRNA gene was amplified using primers 338F (5′-ACTCCTACGGGAGGCAGCA-3′) and 806R (5′-GGACTACHVGGGTWTCTAAT-3′), followed by paired-end (PE) 250 sequencing on the Illumina NovaSeq 6000 platform. Slightly over 3 million raw read pairs were obtained and processed with QIIME2 [31]. Taxonomy was assigned with a Bayes classifier on the SILVA-138 database [32], and 1713 ASVs were derived after filtering. Differences in microbial community composition were evaluated by principal-coordinate analysis (PCoA) based on Binary Jaccard distances calculated using the vegdist function in the vegan R package. Genus-level differential abundance via ANCOM-BC2 [33] and alpha diversity were assessed with phyloseq. Genera present in <10% of samples and samples with <1000 reads were excluded. Functional potential was inferred using PICRUSt2(SC) [34,35] to obtain the unstratified MetaCyc pathway and KEGG Ortholog abundances, of which 11 of 519 and 243 of 8574 candidates (respectively) met criteria (*p* < 0.05, absolute change > 2). The KEGG Ortholog candidates were additionally restricted to the upper 50% by overall mean abundance, with the 5 highest-ranked vehicle-enriched and 5 capsaicin-enriched candidates plotted. The PICRUSt2 inferences are presented to facilitate follow-ups on interesting leads, but these predictions are not confirmed mechanisms and require experimental functional validation.

### 2.9. Metabolomics

Faecal samples from 3 to 4 mice were pooled from the capsaicin and vehicle cohorts and sent to BMK for untargeted metabolomics profiling. A total of 7626 and 9715 features were initially detected in negative and positive ion modes, respectively, and principal component analysis was performed for the 5 capsaicin, 5 vehicle, and 3 QC samples with MetaboAnalyst [36]. Filtering for differentially abundant metabolites (*p*-value < 0.05, fold change ± 20%) yielded 864 candidates, and using BMK’s private and public databases, 587 successfully annotated differential metabolites were then obtained. Additionally, the machine learning random forest algorithm (ntree = 500) was applied to all features to prioritise important metabolites by computing a Mean Decrease Accuracy (MDA) value for each metabolite. A volcano plot was generated for the 587 annotated differential metabolites, displaying log2 Fold Change, –log10 P, and point size proportional to MDA. Subsequently, metabolite set enrichment analysis was conducted based on 139 KEGG pathways integrating host (human) and gut bacteria metabolism.

### 2.10. Serum Lipids Assay

Vehicle- or capsaicin-supplemented (~10 mg/kg/day) TRPV1 KO mice were maintained on HFD for at least 3 weeks [37,38]. A standard oral lipid tolerance test was performed after an 18 h fast. In brief, each animal was administered olive oil (Sigma-Aldrich O1514) at a dose of 10 μL/g body weight via oral gavage. Blood was collected from the tail vein of each mouse immediately prior to (baseline), and at 1, 2, and 4 h after gavage. All blood samples were incubated at room temperature for 30 min and then centrifuged at 1160× *g* for 15 min at 4 °C. The upper serum layer was carefully collected for analysis for triglycerides (Solarbio, Beijing, China, BC5945) and free fatty acids (Solarbio BC0595) following the manufacturers’ protocols [39].

### 2.11. Statistics and Software

General statistical analyses and data visualisation were performed using GraphPad Prism (version 10) and R (version 4). Illustrations were created using BioRender. Randomisation or alternation of mice, measurements, or cage locations was done whenever applicable to minimise confounders. Statistical tests were two-sided and detailed in figure captions: paired one-way ANOVA, unpaired Student’s or Welch’s *t*-tests or Mann–Whitney U tests were used as appropriate. Data are presented as mean ± SEM, except box plots which represent median, 1st and 3rd IQR, with 1.5× IQR whiskers. Statistical significance was defined as *p* < 0.05.

### 2.12. Methodological Limitations

Broad-spectrum antimicrobial treatment is a mainstream strategy in microbiome research, but it may have selective impact on community composition (e.g., bacterial lineages) and off-target effects on host physiology (e.g., liver, gut wall), potentially complicating the interpretation of the microbiome depletion. Hence, conducting complementary studies in germ-free animals and performing faecal microbiota transplantations are avenues for future studies. Notably, as this study highlights the utility of a host receptor knockout experimental platform, we used a male mouse model system for consistency with earlier studies. However, future studies may investigate potential sex differences (e.g., vs. females) or species differences (e.g., vs. primate models), or obesity model dynamics (e.g., vs. longer periods).

## 3. Results

### 3.1. Capsaicin Ameliorates Weight Gain Even in the Absence of Host Receptor

To disentangle the diet–microbiome interactions from the direct diet–host TRPV1 mechanisms, we examined capsaicin’s effect in capsaicin-insensitive hosts. We first established that the TRPV1 knockout (KO) mice were indeed insensitive to capsaicin [19]. As capsaicin’s activation of the host receptor induces a burning sensation, wildtype mice display normal aversion to capsaicin in a dose-dependent manner. In contrast, capsaicin receptor knockout animals consume capsaicin-laced water as if it were plain water (Figure 1A). We then supplemented cohorts of TRPV1 KO mice with capsaicin or vehicle in their drinking water and subjected them to a high-fat diet to induce obesity. As expected, high-fat diet induced dramatic weight gain in the control mice. Remarkably, capsaicin attenuated high-fat diet-driven weight gain, even in animals lacking the capsaicin host receptor (Figure 1B). In parallel, supplementation of capsaicin also improved glucose tolerance compared to vehicle controls (Figure 1C–E), consistent with earlier reports suggesting that capsaicin improves overall metabolic parameters (see Section 4).

We reasoned that the resistance to the dramatic weight gain upon high-fat diet administration may stem from capsaicin-induced changes in either energy intake or energy expenditure (or both). Hence, as a proxy for energy intake, we investigated whether there are differences in high-fat diet consumption in capsaicin-supplemented versus vehicle-supplemented mice. Our findings indicate that capsaicin supplementation does not significantly modify food intake (Figure 1F), even with sensitive daily measures (Figure 1G). Importantly, capsaicin supplementation also did not dampen the animal’s innate and learned hedonic preferences for the high-fat diet (Figure 1H,I). Next, we then reasoned that if the resistance to obesity was not caused by decrease in food consumption, an increased metabolism may underlie the reduced weight gain. Hence, we conducted indirect calorimetry on mice under a diet-induced obesity model. Intriguingly, we found no differences in daily host energy expenditure between capsaicin- and vehicle-supplemented mice (Figure 1J).

### 3.2. Microbiome Mediates Capsaicin’s Effect on Host Health

**Figure 2:**
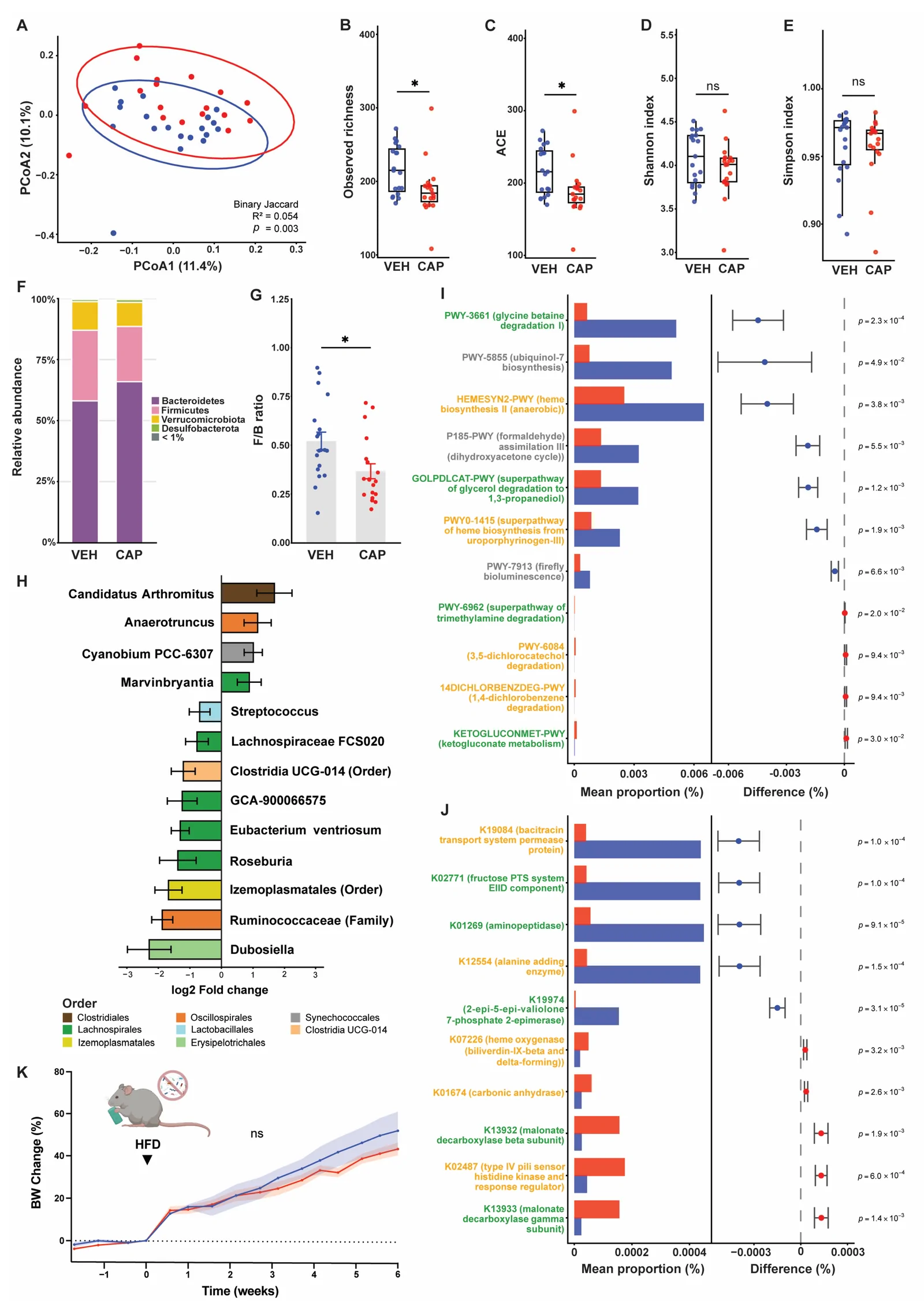
**Microbiome mediates capsaicin’s effect on host health.** (**A**) Beta-diversity principal coordinate analysis (PCoA) plot with 95% confidence ellipses of faecal microbiome from capsaicin-supplemented (red, *n* = 19) and vehicle-supplemented (blue, *n* = 19) mice, showing significant separation (Binary Jaccard, *p* = 0.003, PERMANOVA). (**B**–**E**) Alpha-diversity box plots of microbiomes from mice treated with capsaicin (red) versus vehicle (blue). Significant reduction was observed in richness metrics (in (**B**), *p* = 0.018; in (**C**), *p* = 0.016, Welch’s *t*-test), but not in evenness-dependent diversity metrics (in (**D**): *p* = 0.194; in (**E**): *p* = 0.883, Welch’s *t*-test), suggesting that rare taxa were lost in the capsaicin group, while the core structure involving dominant taxa remained relatively unchanged. (**F**) This point represents the average phyla composition of vehicle-supplemented and capsaicin-supplemented mice, illustrating dominance of Bacteroidetes and Firmicutes in gut microbiome. (**G**) Firmicutes to Bacteroidetes (F/B) phyla ratio is lower in the capsaicin group (*p* = 0.015, Welch’s *t*-test), suggesting better metabolic heath than the vehicle group. (**H**) ANCOM-BC2 analysis reveals 13 differentially abundant genera (*p* < 0.05) between microbiomes from capsaicin versus vehicle treatments. Relative abundance counts are presented in Supplementary Figure S1B–N. (**I**,**J**) PICRUSt2 reveals 11 significantly changed MetaCyc-predicted pathways in (**I**) (|fold change| > 2, *p* < 0.05, Mann–Whitney U test). Bar plots display the mean proportion of each pathway in the capsaicin (red) or vehicle (blue) group. Dot plots show the difference in the mean proportions. In parallel, top 5 enriched and top 5 depleted KEGG ortholog predictions are illustrated in (**J**) (*p* < 0.05) in a similar manner. Collectively, several pathways influence nutrient processing (green), community homeostasis (yellow), and other domains (grey). (**K**) Body weight changes upon transition to HFD are plotted. In microbiome-depleted mice, compared to control (blue, *n* = 4), capsaicin supplementation (red, *n* = 6) did not attenuate weight gain (*p* = 0.41, two-way ANOVA, see also Supplementary Figure S3C), in contrast to capsaicin’s robust effect observed in standard microbiome animals (see Figure 1B), demonstrating that the microbiome is the essential mediator of capsaicin’s effect. Data is presented as mean ± SEM except in box plots as median ± IQR; ns, not significant; * *p* < 0.05.

Collectively, our findings above hint that capsaicin may attenuate weight gain through non-host mechanisms. As the gut microbiome composition is sensitive to dietary elements, we hypothesised that capsaicin, as a supplemented dietary ingredient, will reshape the gut microbiota profile. Hence, we collected faecal samples taken from the TRPV1 KO mice supplemented with capsaicin in their drinking water and conducted 16S rRNA microbiome profiling [31]. Remarkably, dietary supplementation with just capsaicin, a single chemical ingredient, is sufficient to significantly remodel the gut microbiome (Figure 2A–F). At the phylum level, the ratio of Firmicutes to Bacteroidetes, a general gut health biomarker [40], decreased in capsaicin-treated mice compared to that from vehicle-treated mice, indicating a healthier gut microbiome profile with capsaicin (Figure 2G), in agreement with an earlier study in wildtype animals [16].

To further investigate the compositional shifts in the gut microbiome, we conducted differential abundance analysis through ANCOM-BC2 [33] and uncovered four genera that became enriched with capsaicin, and nine taxa that became less abundant (Figure 2H). Notably, Streptococcus and Lachnospiraceae FCS020 group, involved in sugar fermentation and lipoprotein regulation respectively [41,42], were depleted in capsaicin-supplemented mice (Supplementary Figure S1F,G), while Candidatus Arthromitus, a commensal that restricts lipid absorption via Th17 interactions [43,44], was enriched by capsaicin treatment (Supplementary Figure S1B). As changes in a few genera appear to converge intriguingly on the common theme of nutrient handling and harvest, we extended our analysis with functional inference using the PICRUSt2-SC [34,35]. Based on exploratory inference from the MetaCyc database, capsaicin-supplemented animals were predicted to have lower microbial betaine-degrading capacity (Figure 2I, PWY-3661) [45,46]. Moreover, the KEGG inference suggests poorer carbohydrate utilisation and peptide hydrolysis in capsaicin-treated mice (Figure 2J, K02771, K01269) in conjunction with potentially augmented malonate decarboxylase capacity (Figure 2J, K13932, K13933) [47,48,49,50,51,52]. Taken together, the capsaicin-induced microbiome shift possibly selects for functional pathways that may confer metabolic resilience to an excess of dietary lipids when high-fat diet challenge comes later.

Importantly, if microbial community and pathway remodelling are indeed causally required for capsaicin’s effect against diet-induced obesity, then capsaicin should no longer protect against obesity in mice lacking a gut microbiome. To test this, we depleted the gut microbiome in mice lacking the host receptor for capsaicin and then supplemented the mice with capsaicin or vehicle prior to challenging them with a high-fat diet. Remarkably, capsaicin supplementation no longer protected microbiome-deficient TRPV1 KO mice from obesity: both capsaicin and vehicle cohorts displayed similar, rapid weight gain upon high-fat diet challenge (Figure 2K), in contrast to the dramatic effect we observed in standard microbiome (specific-pathogen-free) animals (Figure 1B), thus demonstrating the necessity of the gut microbiome in mediating capsaicin’s protective effect.

### 3.3. Capsaicin Remodels the Gut Metabolome and Dials Down Fat Absorption

**Figure 3:**
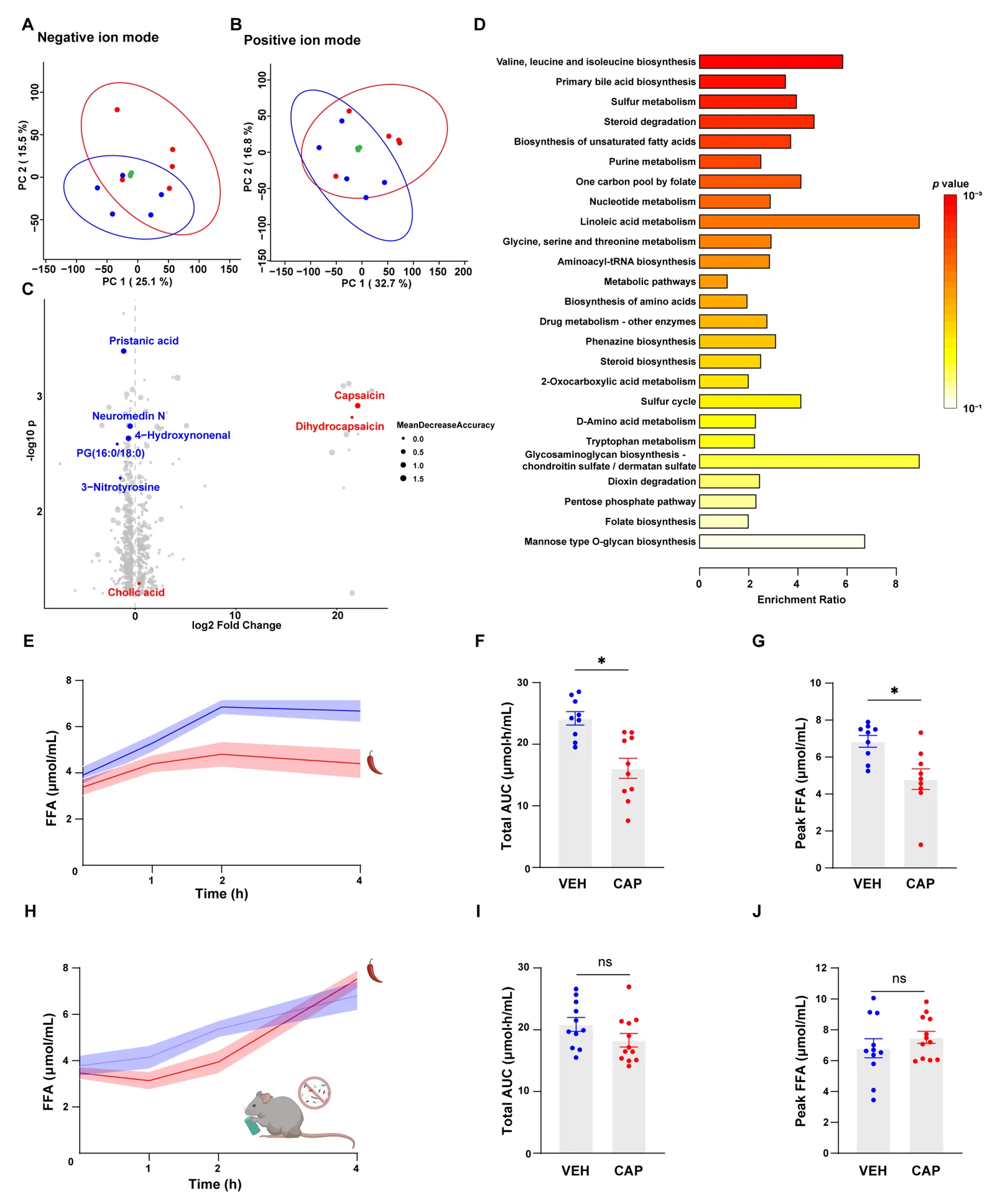
**Capsaicin remodels the gut metabolome and dials down fat absorption.** (**A**,**B**) Principal component analysis (PCA) score plots display metabolite distributions from the faecal metabolome profiles of capsaicin-treated (red, *n* = 5 samples) and vehicle-treated (blue, *n* = 5 samples) mice in negative-ion mode (in (**A**)) and positive-ion mode (in (**B**)), with ellipses representing 95% confidence intervals and QC labelled green. (**C**) Volcano plot of 864 significantly altered faecal metabolites in the capsaicin group versus the vehicle group (fold change ± 20%, *p* < 0.05, Student’s *t*-test), with point size proportional to importance (MDA). Salient compounds enriched with capsaicin treatment (red) or vehicle treatment (blue) are highlighted (see text and Supplementary Figure S2A). (**D**) Top 25 altered metabolic pathways ordered by significance derived from metabolite enrichment analysis of KEGG pathways integrating mammalian host and gut bacterial metabolism. (**E**) Time-course of serum free fatty acid (FFA) concentrations immediately prior to and after oral gavage of olive oil in capsaicin-supplemented (red, *n* = 10) and vehicle-supplemented (blue, *n* = 9) mice. (**F**,**G**) Capsaicin-supplemented mice have a lower total area-under-curve (AUC) of serum FFA during the lipid tolerance test (in (**F**), *p* = 0.00080, Welch’s *t*-test) and lower peak blood FFA concentrations 2 h after intragastric administration of olive oil (in (**G**), *p* = 0.0075, Welch’s *t*-test), indicating dampened absorption of the administered fat. (**H**) Time-course of FFA in capsaicin-supplemented (red, *n* = 11) and vehicle-supplemented (blue, *n* = 11) antibiotic-treated mice. (**I**,**J**) Capsaicin no longer attenuates fat absorption in microbiome-depleted mice, with no change in AUC (in (**I**), *p* = 0.16, Welch’s *t*-test) and in peak FFA (in (**J**), *p* = 0.26, Welch’s *t*-test). Data is presented as mean ± SEM; ns, not significant; * *p* < 0.05.

For capsaicin–microbiome interactions to influence the host’s phenotype, there must be biochemical signals or mediators linking the microbiome to the host. To investigate whether gut microbial metabolites may offer further clues into how capsaicin mechanistically confers protection against obesity, we conducted non-targeted metabolomics from the faecal samples from capsaicin- versus vehicle-treated TRPV1 KO mice. In concordance with the changes in the microbiome community and function, the metabolome from capsaicin-treated animals showed distinct differences from that of control animals (Figure 3A,B). A machine learning random forest classifier, trained on all detected features (7626 in negative-ion mode and 9715 in positive-ion mode), exhibited a modest ability to discriminate between the capsaicin-treated and vehicle groups. The out-of-bag error rate was 40% in both ion modes, corresponding to an overall accuracy of 60%, or 3/5 correctly classified in either group based on confusion matrices. To visually integrate differential abundance, statistical significance, and feature importance, we constructed a volcano plot for the 587 annotated differential metabolites where point size was scaled by importance (positive MDA values) for visualisation (Figure 3C). Not surprisingly, the experimentally administered capsaicinoids are markedly elevated in the gut metabolome of the capsaicin group (Supplementary Figure S2B,C). Differential analysis also revealed that obesity-associated metabolites were markedly reduced (see Section 4, and Supplementary Figure S2D–G) [53,54,55,56,57]. Interestingly, neuromedin N, a neuropeptide that facilitates fatty acid absorption in the intestines [58,59], was decreased in the capsaicin group (Supplementary Figure S2H), hinting at its possible role in mediating how capsaicin protects against diet-induced obesity.

Enrichment analysis of metabolite sets further confirmed profound reconfigurations across key bacterial and host metabolic modules, several of which are associated with nutrient processing. Notably, these include branched-chain amino acid metabolism, linoleic acid metabolism, purine metabolism, unsaturated fatty acid biosynthesis, and primary bile acid biosynthesis (Figure 3D) [60,61,62,63,64,65]. For example, the primary output of host bile acid synthesis is the secretion of cholic acid into the intestines to facilitate dietary lipid absorption. As cholic acid is typically co-absorbed with dietary lipids in the intestines, an excess of excreted cholic acid in the gut metabolome (as observed for the capsaicin group, Supplementary Figure S2I) may suggest blunted lipid absorption in the small intestines, in addition to alterations in secondary bile acid processing by the gut microbiome. Collectively, these salient changes strongly suggest that fat absorption and lipid metabolism are profoundly altered in the capsaicin-supplemented mice.

Motivated by the hints from the microbiome and metabolomics profiling, we hypothesised that the gut microbiome and metabolome reshaped by capsaicin may alter lipid processing to dial down fat absorption. If this were the mechanism by which capsaicin confers resistance to diet-induced obesity, then capsaicin administration should not suppress weight gain for mice on a standard low-fat diet since fat is not in excess. Indeed, while capsaicin supressed weight gain for mice fed a high-fat diet (Figure 1B), capsaicin had no such effect for mice kept on low-fat diets (Supplementary Figure S1A).

To functionally validate whether fat absorption is decreased in capsaicin-treated mice, we conducted an oral fat tolerance test by providing capsaicin- or vehicle-supplemented mice with a bolus of olive oil and tracking the lipid levels in their blood thereafter. If capsaicin dials down fat absorption, we would expect less of the fat bolus to enter the bloodstream from the intestines. Remarkably, that was what we observed as capsaicin-treated mice had milder area-under-curve and lower peak of serum fatty acids concentrations in the two hours after the intragastric administration of fat (Figure 3E–G). Notably, capsaicin-treated mice have lower fasting blood triglycerides, which also suggests potentially improved fat metabolism (Supplementary Figure S2J–L). Taken together, our in vivo assay demonstrates that capsaicin indeed attenuates fat absorption independent of host receptor signalling, confirming our findings from the capsaicin-treated microbiome and metabolome.

Finally, we reasoned that if microbiome interactions are causally required for capsaicin’s effect on fat absorption, then capsaicin supplementation should no longer dampen fat absorption in mice lacking a gut microbiome. To test this, we depleted the gut microbiome in host receptor knockout mice (Supplementary Figure S3) and then conducted the oral fat tolerance test similarly as described above. Incredibly, no significant decrease in serum fatty acids (Figure 3H–J) or triglycerides (Supplementary Figure S2M–O) was observed with or without capsaicin, demonstrating that without an intact microbiome, capsaicin was no longer able to attenuate the blood lipid excursions upon oral administration of the oil.

## 4. Discussion

Food compounds interact not only with the host, but also with the microbiome. However, reciprocal interactions between the host and microbiome complicate disentanglement of food–host effects from food–microbiome mechanisms. To specifically examine the food–microbiome interaction, we focused on capsaicin, a component of chilli peppers and the most common ingredient in spicy cuisines around the world. Using a capsaicin receptor knockout host to mitigate confounding host interactions, we uncovered capsaicin-induced gut microbiome and metabolome changes. We envisage that similar approaches involving genetically engineered knockout animals can be broadly applied to known substrates with canonical host mechanisms, to facilitate studies on food–microbiome mechanisms. With capsaicin as an example, our findings demonstrate that capsaicin supplementation protects against obesity via microbiome-mediated mechanisms.

Numerous studies have hinted that incorporation of chilli peppers or capsaicin in diets will lead to microbiome changes [8,11,12,13,14,16,17,25,26], in line with the general consensus in the field that various food or chemical substrates will modify the microbiome [21,22,23,24]. However, the specific necessity of the gut microbiome in transducing capsaicin’s effect against diet-induced obesity has not been demonstrated prior to this study. To determine whether the microbiome causally mediates the observed phenotype, mice were treated with broad-spectrum antibiotics to eliminate the microbiome. Here, we showed for the first time that microbiota depletion abolished the differences in body weight gain between capsaicin-treated and control groups (Figure 2K), demonstrating that the observed effects of capsaicin are microbiome-dependent.

In host receptor-deficient animals, capsaicin suppresses diet-induced obesity but is itself not anorectic (neither appetite-suppressing, Figure 1F,G, nor reward-suppressing, Figure 1H,I) or thermogenic (Figure 1J). Indeed, if capsaicin supplementation acts via either of these mechanisms, we should expect to see robust weight suppression (or even weight loss) even in KO animals fed a standard low-fat diet. Evidently, that was not the case (Supplementary Figure S1A). Because we observed that the health benefits of capsaicin supplementation only became apparent upon transition to a high-fat diet (Figure 1B), we reasoned that capsaicin supplementation may alter fat processing.

As we have established that the effects are microbiome-dependent, and microbiome interactions may modulate host nutrient digestion and absorption, we then surveyed for capsaicin’s effect on the microbiome and metabolome in host receptor KO mice. We examined the mice after capsaicin (or control) supplementation, but crucially, before the transition to a high-fat diet, as we were cognisant that a major dietary shift will profoundly perturb the microbiome [22] and likely drown out the nuances of capsaicin’s effect. Incredibly, we uncovered that a single ingredient (capsaicin) was sufficient to modestly reconfigure the microbiome taxonomic profile (Figure 2A–H), its functional pathways (Figure 2I,J), and the gut metabolome (Figure 3A–D). This illustrates that although overall diet types and compositions (e.g., processed foods, Mediterranean diet, etc.) are one of the primary drivers that shape our gut microbiome profile, the addition or removal of a single substrate can also significantly reconfigure the microbiome to contribute measurable functional outcomes in controlled settings. In retrospect, this also explains why capsaicin’s effects have been less conclusive especially in highly heterogenous population studies [5,6]. In this regard, our novel approach of mitigating the canonical host receptor-mediated effects may be applied in future in vivo studies to sensitively examine other fine-grained single chemical–microbiome interactions.

We hypothesised that although capsaicin-treated mice consume the same amount of high-fat diet as controls, an attenuation in fat absorption may explain why the capsaicin group exhibits a resistance to high-fat diet-induced obesity. If this is the case, we should expect to see a lower spike in circulating fatty acids upon lipid administration in the capsaicin group. Indeed, the peak fatty acid concentration and area-under-curve of serum fatty acid after an intragastric oil bolus were significantly lower (Figure 3E–G) in the capsaicin-supplemented group, validating our hypothesis. Importantly, these effects of capsaicin on fat absorption were abolished when we eliminated the microbiome (Figure 3H–J), revealing that, independent of the host TRPV1, capsaicin attenuates fat absorption via microbiome mechanisms.

Biologically, capsaicin has great health potential. However, advocating for capsaicin as a dietary supplement to protect against obesity is less practical because its pungency or spiciness may not be tolerated by many people (just as it is unpleasant to wildtype mice, Figure 1A). Although capsaicin’s action via the host TRPV1 mechanisms contributes promoting negative energy balance (for example, by reducing food intake or increasing caloric expenditure in certain contexts) [2,3,4,7], our findings demonstrate that capsaicin’s burning sensation per se is dispensable for capsaicin’s effect against diet-induced obesity, given that capsaicin receptor-deficient hosts still exhibited resistance to obesity. Instead, our results reveal that capsaicin also acts by reconfiguring the microbiome and resists the obesogenic outcomes from high dietary fat intake by dialling down the absorption of dietary fat. Since that is the case, it may be possible that other compounds or dietary habits may produce similar changes in the gut microbiome profile as that induced by capsaicin, and thereby also confer to the host similar resistance against obesity. Such an alternative compound, for example, may essentially also function as a useful prebiotic (without the burning spiciness) that also dials down fat absorption by tweaking the microbiome. Our work indicates that this line of inquiry may now be an exciting next step in the domain of microbiome and obesity sciences.

Interestingly, capsaicin treatment decreases the diversity (richness) of the gut microbiome, indicating that it may selectively deplete certain taxa. Capsaicin’s antimicrobial effects appear predominantly in Firmicutes (Figure 2H, 12 of 13 differentially significant taxa are firmicutes), and the loss of the targeted taxa was sufficient to induce functional shifts that influence microbial community homeostasis: notably in pili signalling, pH balance, heme utilisation, and capacity for antibiotic resistance (Figure 2I,J). While capsaicin’s specific molecular mechanism of action on microbes remains elusive, the phyla selectivity and altered functions may guide future investigations in this area.

Another important next line of inquiry that surfaced is to define the microbial substrate that links the microbiome community changes to the reduction in fat absorption. Our metabolomics approach contributed a list of candidate metabolites—signals that act at the interface between the microbiome and the host, and we anticipate that our list may prove valuable for future research that aims to uncover the microbial-derived molecule that regulates host fat absorption. Notably, based on the decrease in neuromedin N in the capsaicin group (Supplementary Figure S2H), we may speculate that the reduction in this peptide may indeed causally transduce the lipid metabolism effect from the microbiome to the host. Dialling into neuromedin N’s natural pathway for modulating dietary lipid absorption may provide a transformative handle in mitigating fat overconsumption and associated metabolic comorbidities.

Finally, availing improved research tools accelerates scientific advancement. We have designed an innovative rodent food hopper that enables us to accurately measure food intake of individual mice daily, with the sensitivity and resolution that was not afforded by a conventional cage food hopper (compare Figure 1F vs. Figure 1G). In particular, reliable quantification of high-fat diet consumption in standard mice cages has been a longstanding challenge. We hope that contributing the engineering procedure and blueprints (see Supplementary Materials) will benefit other labs and the broader research community also keen on phenotyping the nuances of food consumption behaviour. Our food hopper system uses accessible and economical materials such as stainless-steel sheets and acrylic sheets, has a modular design for scalability, and can be assembled in most research institutions that have access to a makerspace/workshop with a laser cutter or are otherwise easily outsourced.

## 5. Conclusions

Distinguishing direct host-receptor pathways from indirect, microbiome-mediated effects of dietary bioactives remains a fundamental challenge in nutritional science. Using capsaicin as a proof-of-principle food compound, we addressed this complexity by employing a high-fat diet (HFD)-induced obesity model in capsaicin-insensitive, TRPV1 knockout mice. Remarkably, dietary capsaicin supplementation significantly attenuated weight gain and improved glucose tolerance even in hosts lacking the canonical capsaicin receptor. This metabolic protection was entirely abolished upon depletion of the gut microbiota, demonstrating that the microbiome is an essential mediator of capsaicin’s anti-obesity effects. Interestingly, while capsaicin did not alter cumulative dietary intake, food preferences, or host energy expenditure, it substantially remodelled the gut microbiome and metabolome in ways that converged on altered nutrient processing, specifically pointing toward a reduction in lipid absorption. We validated this mechanism in vivo via oral fat tolerance challenges, demonstrating that capsaicin-treated mice exhibit significantly blunted postprandial increases in circulating lipid levels—an effect that was also reversed in microbiome-depleted cohorts. Collectively, our findings unmask a nuanced, non-canonical mode of action where capsaicin modulates the gut microbiome to dial down host fat absorption, while establishing a generalisable host-knockout experimental framework for dissecting the interactions among food elements, microbiome, and host. 

## Data Availability

The original contributions presented in this study are included in the article/Supplementary Materials. Further inquiries can be directed to the corresponding author.

## Supplementary Materials

The following supporting information can be downloaded at: https://www.mdpi.com/article/10.3390/nu18213667/s1. ‘Supplementary Figures.pdf’ (Supplementary Figure S1. Capsaicin reshapes composition of gut flora; Supplementary Figure S2. Capsaicin alters gut metabolites and serum triglycerides; Supplementary Figure S3. Antibiotic treatment depletes the microbiome in TRPV1 KO mice); ‘Source Files.zip’ (Supplementary Materials S1 and S2: Machine files for engineering; Supplementary Material S3: Feeder construction blueprint).
