# Supplementary material for: Dietary Capsaicin Protects Against Obesity via a Microbiome-Dependent Mechanism Independent of Host TRPV1 Signalling
Source: Nutrients. 2026 Nov 4;18(21):3667. doi: 10.3390/nu18213667 (PMC13765459; doi:10.3390/nu18213667)
Supplement: Supplementary file 1 [file nutrients-18-03667-s001.zip › Supplementary Figures.pdf]

Supplementary Figures

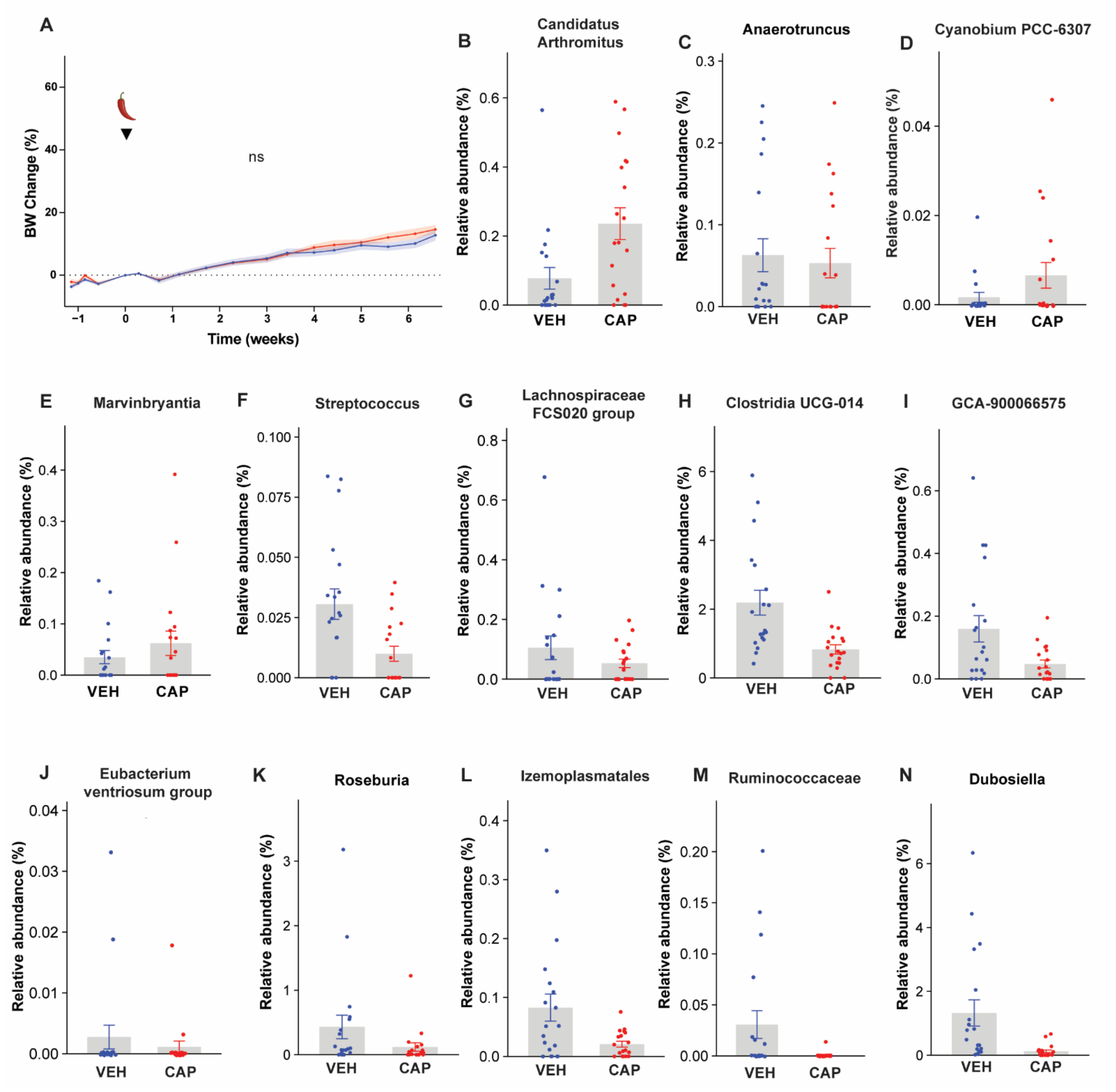

Supplementary Figure S1. Capsaicin reshapes composition of gut flora

(A) Body weight changes upon supplementation with capsaicin (red, *n* = 7) versus vehicle (blue, *n* = 7) in TRPV1 KO mice on a low-fat (11% kcal) diet. In contrast to capsaicin's robust effect on attenuating high-fat (60% kcal) diet-induced weight gain (see Figure 1B), no significant difference was found on a low-fat diet ($p = 0.50$, two-way ANOVA).

(B-N) Bar graphs illustrating relative abundance of genera listed in Figure 2H (ANCOM-BC2), for microbiomes from capsaicin-treated (blue) and vehicle-treated (red) mice.

Supplementary Figures

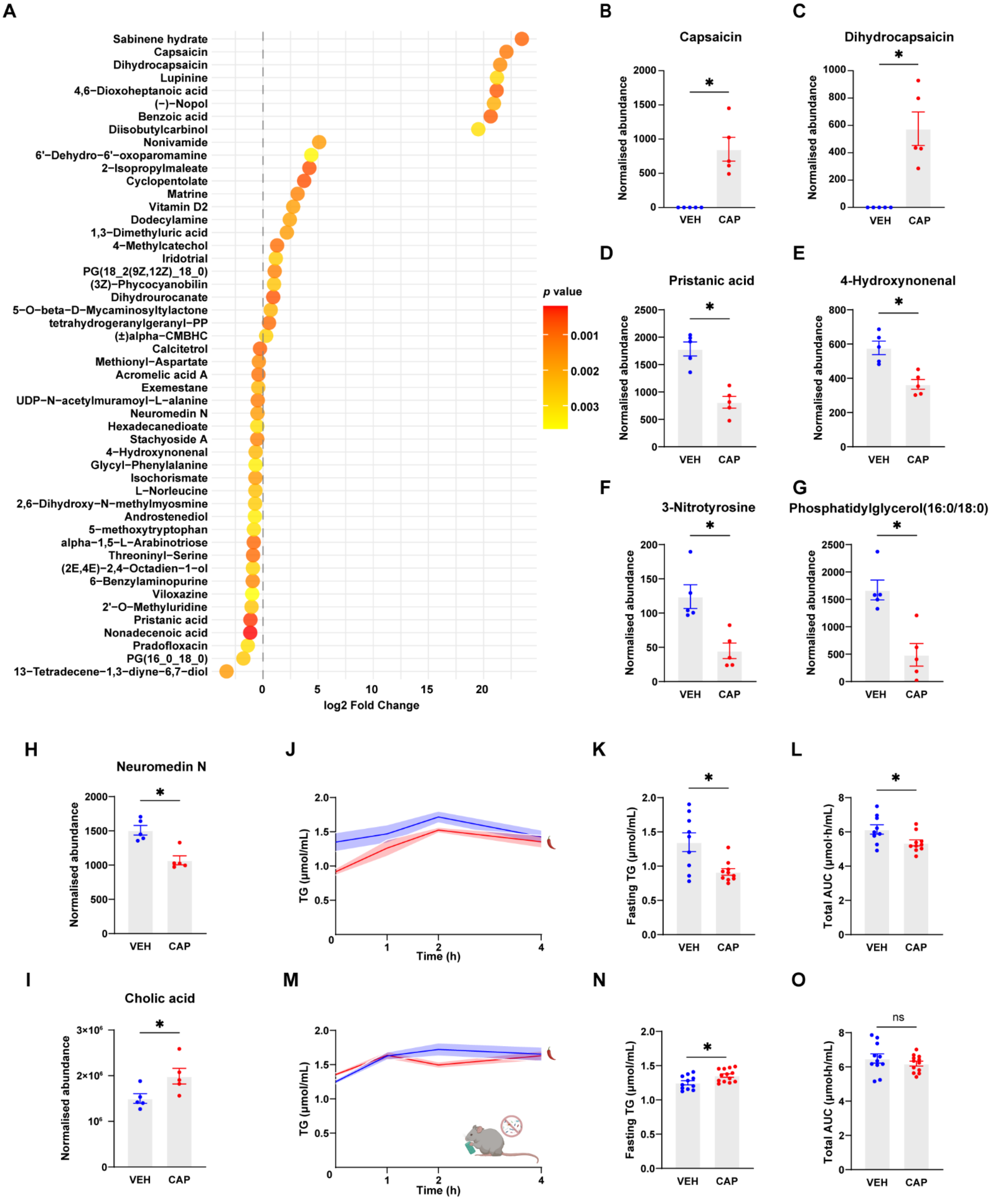

Supplementary Figure S2. Capsaicin alters gut metabolites and serum triglycerides

(A) Bubble plot of top 50 significantly altered faecal metabolites, from Figure 3C, in capsaicin versus control groups, ordered by fold change and coloured by *p* value.

(B-I) Normalised abundance of significantly altered metabolites, highlighted in Figure 3C, in the gut metabolome of mice treated with capsaicin (red, *n* = 5) or vehicle (blue, *n* = 5).

Supplementary Figures

(J) Time-course of serum triglyceride concentrations immediately prior to and after oral gavage of olive oil in capsaicin-supplemented (red, n = 10) and vehicle-supplemented (blue, n = 9) mice.

(K-L) Oral lipid tolerance test reveals that capsaicin-supplemented mice have lower fasting blood triglycerides (in **K**, p = 0.013, Welch's t-test) and a lower total area-under-curve (AUC, in **L**, p = 0.0314, Welch's t-test), indicating improved metabolic health compared to controls.

(M) Time-course of serum triglyceride in capsaicin-supplemented (red, n = 11) and vehicle-supplemented (blue, n = 11) microbiome-depleted mice.

(N-O) Without microbiome, capsaicin no longer lowered fasting blood triglycerides (a slight increase was observed in **N**, p = 0.018, Welch's t-test), and no longer altered the total AUC (in **O**, p = 0.29, Welch's t-test).

Supplementary Figures

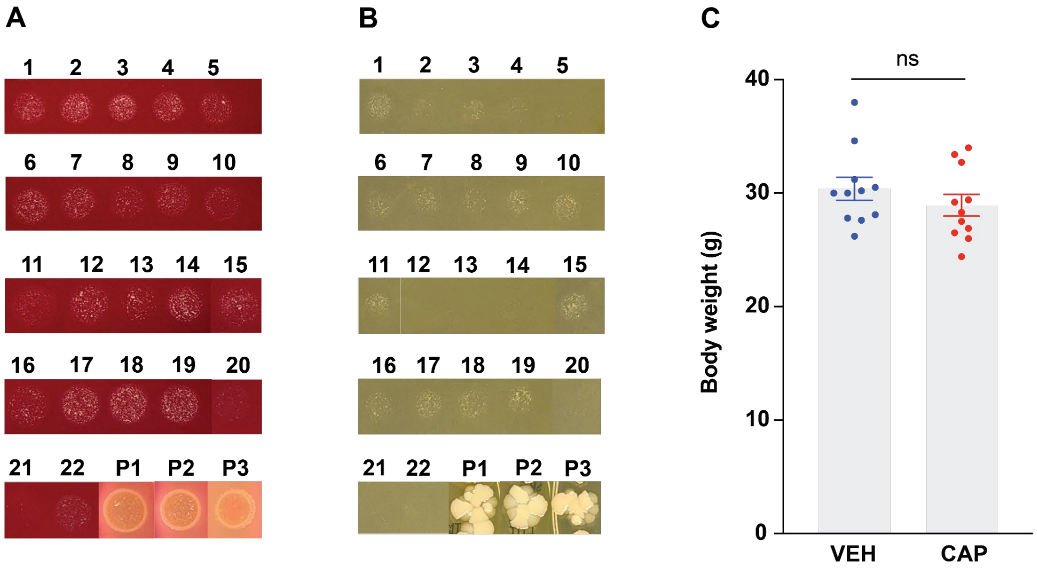

Supplementary Figure S3. Antibiotic treatment depletes the microbiome in TRPV1 KO mice

(A-B) Antibiotic-induced depletion was assessed by drop plating faecal samples (20 mg/ml) from antibiotic-treated mice (n = 22) and non-antibiotic-treated SPF mice (n = 3), on Schaedler (A) and BHI (B) agars in anaerobic and aerobic conditions respectively. Faint circular marks/spots are suspended solids, as samples were homogenised but not filtered to maximize detection sensitivity.

(C) Body weights of antibiotic-treated mice supplemented with capsaicin (n = 11) or vehicle (n = 11) after 3 weeks on HFD (prior to oral lipid tolerance tests in Figure 3H). As expected, capsaicin failed to attenuate obesity in microbiome-depleted animals (p = 0.31, Welch's t-test, see also Figure 2K).
