# Supplementary material for: Dietary Capsaicin Protects Against Obesity via a Microbiome-Dependent Mechanism Independent of Host TRPV1 Signalling
Source: Nutrients. 2026 Nov 4;18(21):3667. doi: 10.3390/nu18213667 (PMC13765459; doi:10.3390/nu18213667)

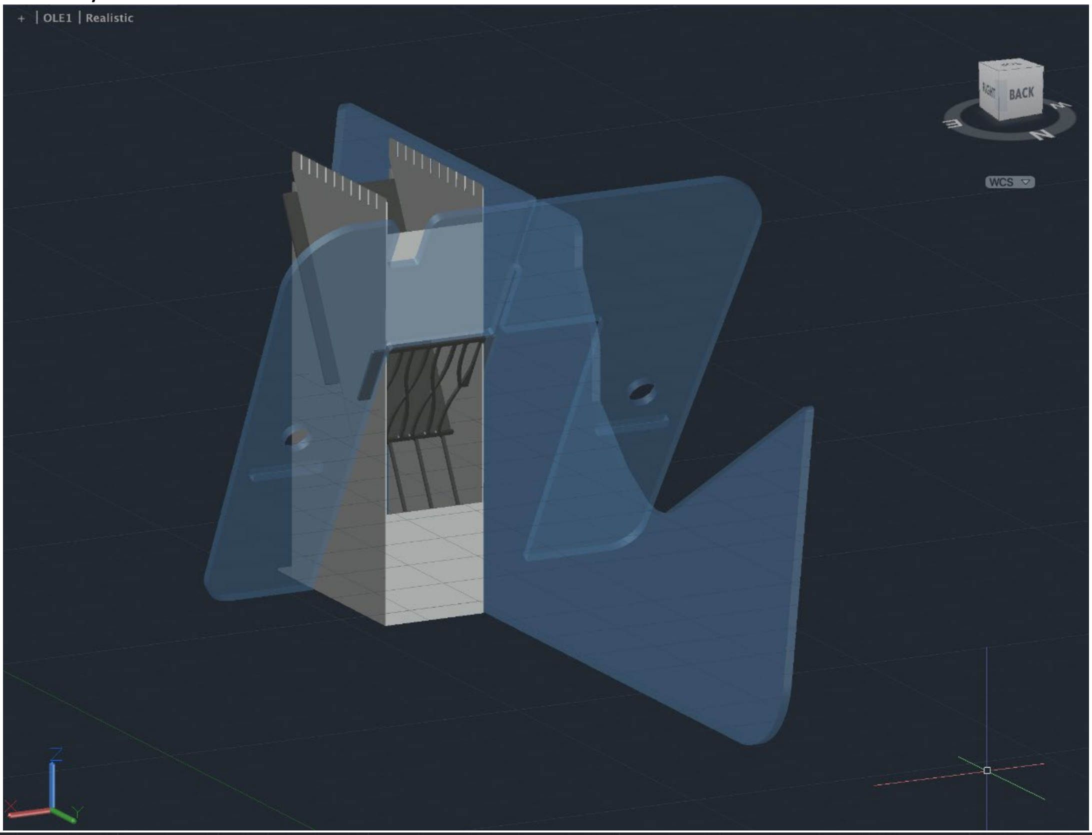
OLE1 | Realistic
BACK
WCS

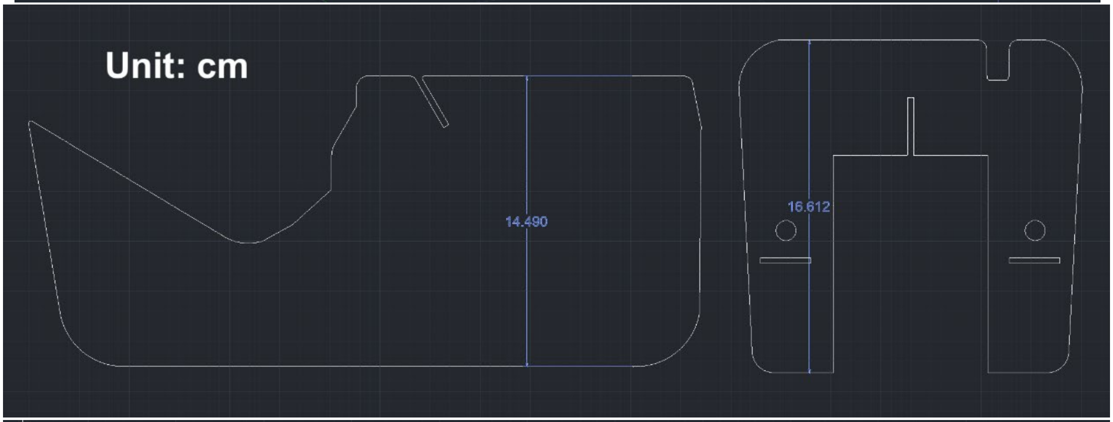
Unit: cm
14.490
16.612

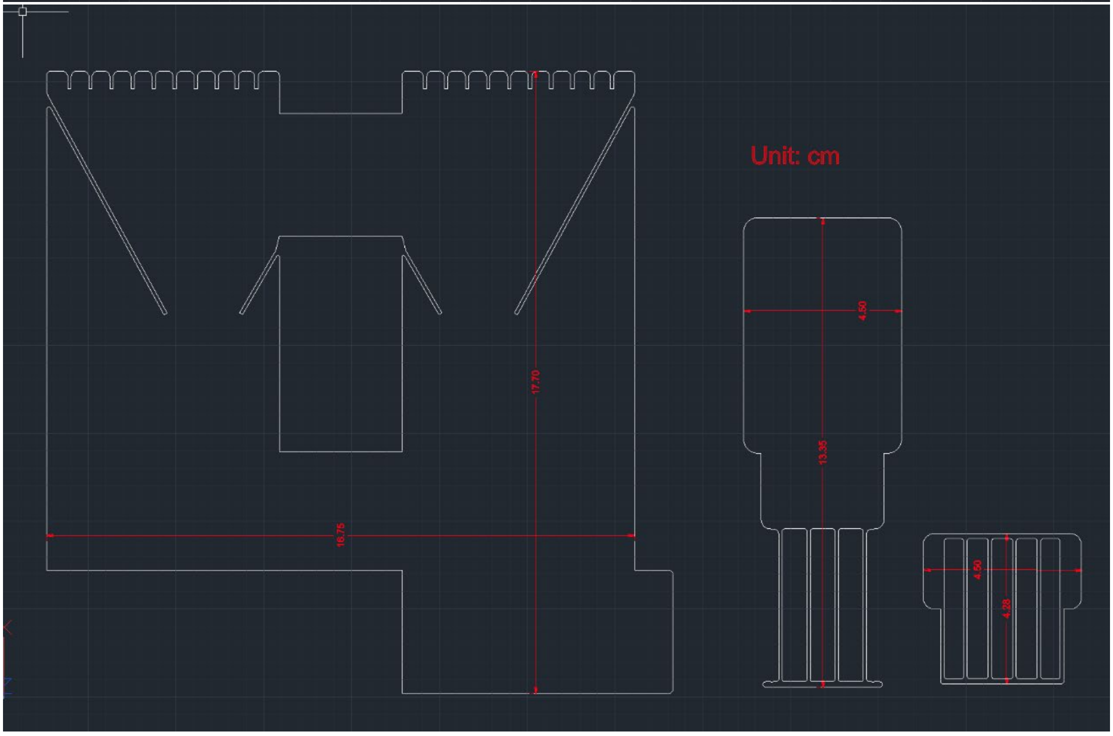
Unit: cm
17.70
16.75
4.50
13.35

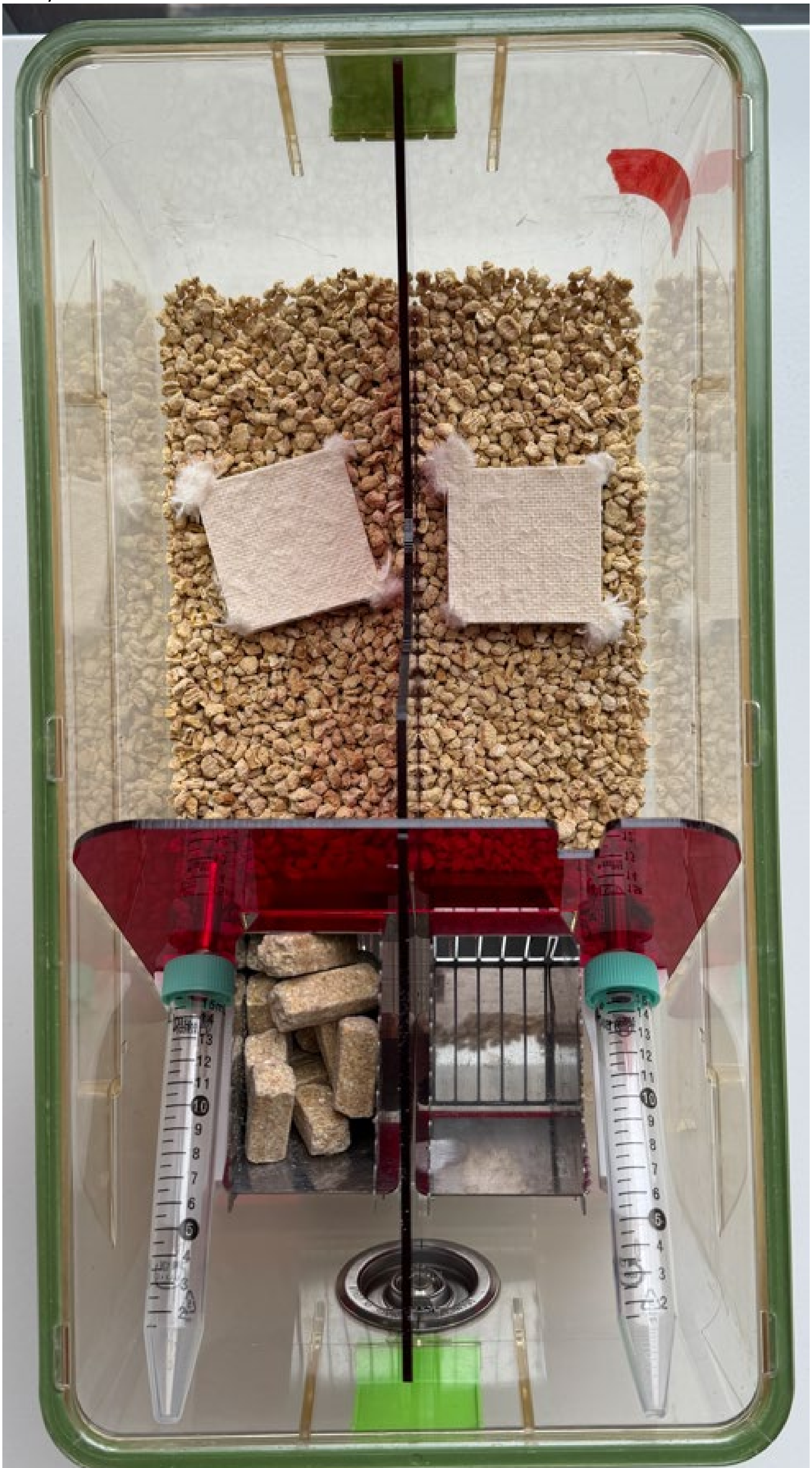

Supplement: Supplementary file 1 [file nutrients-18-03667-s001.zip › Source Files.zip!/Supplementary Material 3 Feeder construction.pdf]
